# The Use of Equine-Assisted Therapy in Patients With Aggression and Agitation Behaviors due to Moderate-to-Severe Dementia: A Case Series

**DOI:** 10.1155/crps/8785490

**Published:** 2025-06-04

**Authors:** Beatriz Pozuelo Moyano, Jean Pierre Schuster, Kevin Swierkosz-Lenart, Leonardo Zullo, Charline Compagne, Caroline Imobersteg, Armin von Gunten, Pierre Vandel

**Affiliations:** ^1^Service of Old Age Psychiatry, Department of Psychiatry, Lausanne University Hospital and University of Lausanne, Prilly 1008, Switzerland; ^2^Leenaards Memory Center, Department of Clinical Neurosciences, Lausanne University Hospital and University of Lausanne, Lausanne 1011, Switzerland

**Keywords:** behavioral and psychological symptoms of dementia, dementia, equine-assisted therapy, older persons

## Abstract

Behavioral and psychological symptoms of dementia (BPSD) are very common, and their management remains challenging. In older people with dementia, equine-assisted therapy (EAT) may be a promising nonpharmacological intervention for the management of BPSD. Here, we present five cases of patients with agitation and aggression due to moderate-to-severe dementia. They had overall two to three sessions of EAT at a frequency of one session per week. We assessed the Neuropsychiatric Inventory Questionnaire (NPI-Q) score before and 1 day after the second EAT session. We observed a discrete reduction in the NPI-Q after the EAT sessions, although not all of the improvements experienced by patients, families, and carers were reflected in the NPI-Q. Future studies should be conducted to assess subjective lived experiences of EAT in patients with moderate-to-severe dementia.

## 1. Introduction

Behavioral and psychological symptoms of dementia (BPSD) refer to a range of noncognitive disturbances that are common in people with dementia. These symptoms include apathy, depression, anxiety, sleep disturbances, agitation, aggression, or psychosis [1]. The prevalence of BPSD is high, as BPSD affect at least 90% of people diagnosed with dementia, who often experience at least one BPSD symptom during the course of their illness [1, 2]. BPSD can be predisposed by various factors such as medical illnesses, medications, unmet patient needs, or pain [3]. People with BPSD are more likely to enter long-term care and have increased morbidity and mortality. BPSD are associated with a faster progression of dementia, and they increase caregiver's burden [1, 4].

Management of BPSD involves both nonpharmacologic and pharmacologic approaches [5]. Among nonpharmacological therapies, animal-assisted activities have emerged as potential treatments [6]. Animal-assisted therapy (AAT) is defined as the use of an animal considered appropriate to work with human care recipients in the treatment of human physical or psychological disorders coordinated by a human professional with in-depth knowledge of the animals involved and who has been formally certified [7].

Animal-assisted interventions involve structured interactions between patients and animals, typically aimed at providing therapeutic benefits [8]. These approaches are heterogeneous and based on different constructs depending on the type of animal used, the specific techniques used, and the training for the carer [8, 9]. AAT can vary and often include multisensory stimulation, physical contact, or play offered in groups or individually [8, 9].

Three systematic reviews have examined the effect of AAT on BPSD. Due to the studies being very small, evidence remains uncertain [8, 10, 11]. A Cochrane systematic review, including randomized controlled trials, cluster randomized trials, and randomized cross-over trials evaluating dog-assisted therapies and equine-assisted therapy (EAT), concluded that AAT, may reduce depressive symptoms in people with dementia, but there is no clear evidence on its effects on different possible outcomes such as social functioning, quality of life, behavior, or physical and cognitive functioning [6, 11].

Yakimicki et al. [8] conducted a systematic review that showed a significant positive impact on social behaviors, physical activity, and dietary intake in people with dementia engaging in AAT. In total,15 studies measured outcomes related to agitation and/or aggression, some of them with quasi-experimental designs with multiple methodological variations. About 9 out of 15 studies reported a significant decrease in agitation and aggression. In terms of social behavior, 11 out of 12 studies showed increased social interaction [8]. A more recent systematic review of 10 randomized controlled trials and cohort studies found positive effects of AAT on depression [10]. The three systematic reviews mainly assessed studies of interventions with dogs, and one only included study of EAT [11].

EAT is a specialized form of AAT that is effective for improving attention and quality of life in children with cerebral palsy or autism [12, 13]. In adult populations, EAT is an effective treatment to improve emotion regulation in patients with substance use disorders [14]. In patients with schizophrenia or schizophrenia-like disorders, EAT is potentially useful to facilitate remission and recovery [15]. EAT has also shown positive results in improving posttraumatic stress disorder symptoms [16].

In people with dementia, EAT has been found to be effective in managing BPSD and improving quality of life [17]. A recent systematic review summarizing the evidence from six studies (four quantitative and two qualitative) of varying study designs of moderate-to-strong quality concluded that EAT had positive effects on social, emotional, and behavioral outcomes, including well-being, social participation, and communication [17].

In the present report, we describe five cases in which people living with moderate-to-severe mixed dementia hospitalized due to agitation or aggression had overall two or three sessions of EAT at a frequency of one session per week.

## 2. Procedure and Case Presentation

The patients described below were admitted to the service of old age psychiatry due to an acute exacerbation of their behavioral disorders, at the time of the EAT intervention. It is important to note that patients also received other interventions (i.e., pharmacological or nonpharmacological treatments in hospital) aimed at improving BPSD. The decision to proceed with the EAT was made after a multidisciplinary discussion of each patient, considering whether there were any physical contraindications. In addition, we considered the patients' preferences for animals in adulthood, after consulting with their families or caregivers. The EAT sessions were held at a farm about a 10-min walk from the hospital. Patients were always accompanied by two trained professionals, one of whom was a physiotherapist who had previously performed a physical assessment evaluating motor skills, gait, and risk for falls. The physiotherapist also provided assistive devices such as walkers or canes when needed to ensure patient safety; these were supplied by the hospital. In cases where walking was not possible, patients were transported by car. EAT sessions involved a patient and a horse or pony, with the patient being introduced to the equine by a specialist therapist. Each patient was assigned a horse or pony, provided with a grooming box. After tactile contact, a walk with the equine took place, allowing the patient to see other farm animals. At the end of the session, the patient fed animal, if possible, as a reward. The frequency of the EAT sessions was once a week, with the patients receiving a total of two or three sessions over 3 weeks. The total duration of each session was 1 h. During EAT, we paid special attention to the safety and welfare of the animals, which is key to EAT approaches [7].

### 2.1. Measures

We carried out a systematic BPSD assessment using the Neuropsychiatric Inventory Questionnaire (NPI-Q) scale before the EAT therapy and 1 day after the second EAT session. NPI-Q assesses the presence of the 12 BPSD also providing an index of the corresponding severity of each symptom and the respective distress it causes to the health career [18].

### 2.2. Case Presentations

#### 2.2.1. Case 1

**Figure 1:**
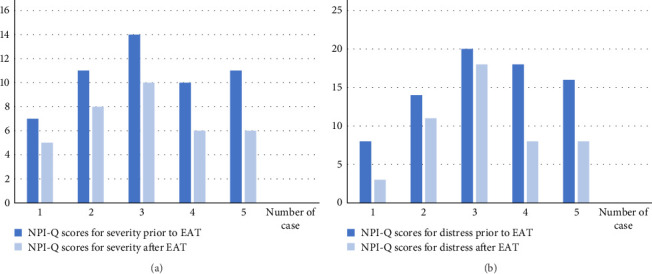
Changes in NPI-Q scores before EAT and 1 day after the second session of EAT (A) NPI-Q scores for severity, (B) NPI-Q scores for distress.

Mrs M was a 58-year-old woman with a history of posterior cortical atrophy (PCA) due to Alzheimer's disease (AD) diagnosed at the age of 56 years. She was admitted to the psychogeriatric service for severe agitation. Her cognitive impairment was too advanced to perform a Montreal Cognitive Assessment (MoCa) [19] or other form of standardized cognitive testing. During her hospitalization, pharmacological treatment (quetiapine, gabapentin, trazodone, and midazolam) was not effective. Mrs M experienced the most common symptoms of PCA, such as severe visuospatial and visuoperceptual alterations, oculomotor apraxia and optic ataxia, and environmental agnosia. The loss of these abilities was associated with high levels of anxiety, which was often associated with aggression. Although she did not show any particular interest in animals, she was able to attend to EAT sessions, and pet the animals calmly. We did not observe any agitation in the hours following treatment. The NPI-Q total scores decreased slightly after EAT (Figure 1).

#### 2.2.2. Case 2

M A was an 82-year-old patient admitted to our hospital with a history of AD and agitation. At the time of EAT, he was limited in his activities of daily living (ADLs 2/6 [20]) and was unable to perform any instrumental ADLs. His MoCa test score was 5/30. During EAT, he communicated verbally with carers. He had two sessions of EAT and experienced a reduction in NPI-Q scores (Figure 1).

#### 2.2.3. Case 3

M B is a 69-year-old patient with a history of dementia of mixed etiology (neurodegenerative and vascular) and epilepsy. He was admitted to the hospital with advanced dementia (Clinical Dementia Rating [CDR 3] [21]) and a history of several episodes of aggression toward carers and other residents in the nursing home. After several treatment attempts, his behavior improved with a pharmacological treatment comprising carbamazepine, quetiapine, levetiracepam, as well as electroconvulsive therapy (ECT). Despite the above treatment, he still had episodes of verbal aggression, particularly in interactions with other patients or carers. He benefited from EAT, which he tolerated very well and reduced aggression and the NPI-Q scores (Figure 1). During the first session, he spontaneously caressed the horses with gentle strokes, verbalized that he enjoyed the activity, was calm, smiled, and was able to have interactions with the carers and other patients. During the next sessions, he smiled from the moment the EAT carer arrived. He was able to thank the carers after the two sessions. He did not have an aggressive episode during the sessions.

#### 2.2.4. Case 4

M H was a 73-year-old patient with mixed dementia. After an infection with severe acute respiratory syndrome caused by Coronavirus 2, his condition deteriorated rapidly, resulting in a persistent confusional state with significant fluctuations. He was admitted to hospital with moderate dementia (CDR 2) and presented with agitation and irritability. During the EAT sessions, he was calm and engaged with the carers and the animals. From the second session onward, he was fully oriented in the stables with the horses,; he knew where to find the utensils to clean them. Despite episodic memory problems and executive difficulties, he was able to find the right tools to clean horses in consecutive sessions. In consecutive sessions, he remembered the horse with which he had engaged with in the previous session. After the two sessions, we observed a reduction in the NPI-Q (Figure 1).

#### 2.2.5. Case 5

M C was a 75-year-old man with AD. He was hospitalized due to agitation and irritability. He had advanced cognitive impairment, with his speech being disorganized and uninformative. He was dependent on all ADLs and required constant guidance. A treatment with neuroleptics was attempted but had to be discontinued due to intolerance with frequent falls and significant extrapyramidal symptoms. The patient received two sessions of EAT, which led to an overall improvement, with Mr C being able to express his enjoyment for the activity and his desire to continue engaging. After the sessions, we observed a reduction in the NPI-Q scores (Figure 1).

## 3. Discussion

This case series reports on a group of patients hospitalized due to severe irritability, aggression, or agitation, all living with advanced dementias of various etiologies. The patients were heavily dependent in their ADLs, with language impairments impeding participation in conventional psychotherapeutic treatments. All patients were treated pharmacologically and some had undergone ECT [22]. However, these treatments were only partially effective and limited in some instances by the emergence of significant side effects.

One of the main findings is the feasibility of EAT with people with dementia hospitalized in the acute phase of their illness. Despite the significant cognitive and, in some cases, physical limitations of these patients and high levels of BPSD that required hospitalization, we observed that EAT was a therapy well accepted by both patients and health professionals.

In our cases, we observed a global reduction in the NPI-Q score after EAT (Figure 1). However, it is important to note that the BPSD of these patients may also have benefited from concurrent interventions, including global inpatient care and possible placebo effects. At that, caregivers observed,in all cases, an awareness of the activity being performed, which had not been previously noted for other activities. This included improved social behavior, including gestures and verbal expressiveness. Patients were also able to express their satisfaction with this type of therapy.

Our case series and experience suggest that EAT can be safe when an appropriate infrastructure is in place, and the intervention is delivered by an experienced team. It has been well received by both patients and carers, with a positive impact on patients and symptoms of agitation and aggression in dementia. The current setting benefited from the unique proximity of the EAT center to the hospital, which facilitated efficient participation. However, feasibility may be more complex in other settings due to the frailty of this patient population and the logistical, spatial, and financial demands associated with this type of intervention. In other hospitals, the feasibility and safety of this will depend on the accessibility of adapted transport and the availability of a suitably trained multidisciplinary team. Our experience suggests that where these conditions are met, EAT can be considered a viable option for people with dementia. Agitation and aggression are complex and frequent behaviors, not linearly associated with the severity of dementia [23]. This suggests multiple etiopathogenic pathway toward clinical BPSD, among the many, a lack of stimulation and a lack of engagement in activities [2]. Thus, agitation or aggression can be related to the therapeutic interaction between carers and patients, which is crucial in preventing the BPSD. Unfavorable interactions between caregivers and people with dementia undermine personhood and lead to unfulfilled social and psychological needs [24]. Communication between carers and people with dementia in hospitals or nursing homes tends to be directive and dominated by instructions [25]. However, during an outdoor activity such as EAT, other types of communication can be experienced inducing normalized communicative exchanges and limiting the “fragile patient” perception associated with the care environment. These interactions can facilitate the carers' awareness and consideration of other very important factors, such as engaging in activities. According to the need-driven, dementia-compromised behavior theory [26], behaviors commonly classified as disruptive in dementia, are actually the result of unfulfilled needs. These can be physical (pain alleviation, thirst, hunger, constipation, or infection or their treatments), emotional (communicational deficits, discomfort, or pathological interaction), or recreational (absence or overflow of stimulating activities). There is evidence showing that well-dosed recreational activities have positive effects for BPSD [27]. EAT may contribute to fulfilling the need for engaging in activities and interacting with others. Similarly, we observed significant improvement in our patients when measuring the change in the NPI-Q score after EAT. However, only some of the improvements experienced by patients, families, and carers were reflected in the NPI-Q.

Sometimes aggression or agitation may be directly related to a reversible condition of a somatic etiology or pain, but in other cases, they may be related to premorbid personality, or loss of pleasant activities, or engaging in social interactions with others [28–30]. Our series of cases is relevant because it supports existing but limited systematic reviews on the positive effects of EAT for people with dementia. In our case series, most patients lived with severe dementia and had significant cognitive impairment. Here, we illustrate that the severity of dementia is not a limitation for these therapies. Furthermore, compared to previous studies, our patients had only two to three EAT sessions. Although this is a limitation of this case series, it shows that a single session can be useful for patients with aggression or agitation at least in the short run.

## 4. Conclusion

The existing literature emphasizes the need for safer and more effective alternatives to pharmacological interventions for BPSD in people with advanced dementia. This case series shows that EAT may have potential as a nonpharmacological treatment option. Future studies assessing lived experiences and feasibility of EAT for people with advanced dementia and BPSD are needed. Another area that warrants further investigation is the differential indication of EAT for different types of BPSD according to their clinical presentation or their etiopathogeny.

## Data Availability

The data that support the findings of this study are available from the corresponding author upon reasonable request.

## References

[1] Ayhan Y., Yoseph S. A., Miller B. L. (2023). Management of Psychiatric Symptoms in Dementia. *Neurologic Clinics*.

[2] Tible O. P., Riese F., Savaskan E., Von Gunten A. (2017). Best Practice in the Management of Behavioural and Psychological Symptoms of Dementia. *Therapeutic Advances in Neurological Disorders*.

[3] Bessey L. J., Walaszek A. (2019). Management of Behavioral and Psychological Symptoms of Dementia. *Current Psychiatry Reports*.

[4] Schuster J. P., Santos Z., Von Gunten A. (2023). Polysemy of Agitation in Dementia. *Revue Médicale Suisse*.

[5] Savaskan E., Georgescu D., Becker S. (2024). Recommendations for the Diagnostic and Therapy of Behavioural and Psychological Symptoms of Dementia (BPSD). *Praxis*.

[6] Lai N. M., Chang S. M. W., Ng S. S., Tan S. L., Chaiyakunapruk N., Stanaway F. (2019). Animal-Assisted Therapy for Dementia.. *Cochrane Database of Systematic Reviews*.

[7] Jegatheesan B. (2014). IAHAIO White Paper.

[8] Yakimicki M. L., Edwards N. E., Richards E., Beck A. M. (2019). Animal-Assisted Intervention and Dementia: A Systematic Review. *Clinical Nursing Research*.

[9] Schuster J. P., Pozuelo Moyano B. (2024). Animals in Nursing Homes? (Des Animaux en EMS?). *La gazette médicale*.

[10] Batubara S. O., Tonapa S. I., Saragih I. D., Mulyadi M., Lee B.-O. (2022). Effects of Animal-Assisted Interventions for People With Dementia: A Systematic Review and Meta-Analysis. *Geriatric Nursing*.

[11] Dabelko-Schoeny H., Phillips G., Darrough E. (2014). Equine-Assisted Intervention for People With Dementia. *Anthrozoös*.

[12] Ahn B., Joung Y.-S., Kwon J.-Y. (2021). Effects of Equine-Assisted Activities on Attention and Quality of Life in Children With Cerebral Palsy in a Randomized Trial: Examining the Comorbidity With Attention-Deficit/Hyperactivity Disorder. *BMC Pediatrics*.

[13] Pantera E., Froment P., Vernay D. (2022). Does Hippotherapy Improve the Functions in Children With Cerebral Palsy? Systematic Review Based on the International Classification of Functioning.. *Journal of Integrative and Complementary Medicine*.

[14] Souilm N. (2023). Equine-Assisted Therapy Effectiveness in Improving Emotion Regulation, Self-Efficacy, and Perceived Self-Esteem of Patients Suffering From Substance use Disorders. *BMC Complementary Medicine and Therapies*.

[15] Jormfeldt H., Carlsson I.-M. (2018). Equine-Assisted Therapeutic Interventions Among Individuals Diagnosed With Schizophrenia. A Systematic Review. *Issues in Mental Health Nursing*.

[16] Palomar-Ciria N., Bello H. J. (2023). Equine-Assisted Therapy in Post-Traumatic-Stress Disorder: A Systematic Review and Meta-Analysis. *Journal of Equine Veterinary Science*.

[17] Sebalj M., Lakhani A., Grindrod A., Stuckey R. (2024). Equine-Assisted Services for People Living With Dementia: A Systematic Review. *Alzheimer’s Research & Therapy*.

[18] Kaufer D. I., Cummings J. L., Ketchel P. (2000). Validation of the NPI-Q, a Brief Clinical Form of the Neuropsychiatric Inventory. *The Journal of Neuropsychiatry and Clinical Neurosciences*.

[19] Julayanont P., Nasreddine Z. S., Larner A. J. (2017). Montreal Cognitive Assessment (MoCA): Concept and Clinical Review. *Cognitive Screening Instruments*.

[20] Edemekong P., Bomgaars D., Sukumaran S., Schoo C. (2023). *Activities of Daily Living*.

[21] Berg L. (1984). Clinical Dementia Rating. *British Journal of Psychiatry*.

[22] Swierkosz-Lenart K., Mall J. F., Von Gunten A. (2019). Interventional Psychiatry in the Management of Behavioural and Psychological Symptoms of Dementia: A Qualitative Review. *Swiss Medical Weekly*.

[23] Livingston G., Barber J., Marston L. (2017). Prevalence of and Associations With Agitation in Residents With Dementia Living in Care Homes: MARQUE Cross-Sectional Study. *BJPsych Open*.

[24] Kitwood T. (1997). *Dementia Reconsidered: The Person Comes First*.

[25] Cohen-Mansfield J., Creedon M. A., Malone T., Parpura-Gill A., Dakheel-Ali M., Heasly C. (2006). Dressing of Cognitively Impaired Nursing Home Residents: Description and Analysis. *The Gerontologist*.

[26] Algase D. L., Beck C., Kolanowski A. (1996). Need-Driven Dementia-Compromised Behavior: An Alternative View of Disruptive Behavior. *American Journal of Alzheimer’s Disease*.

[27] Mitchell K., Van Puymbroeck M. (2019). Recreational Therapy for Dementia-Related Symptoms in a Long-Term Care Setting: A Case Study. *Therapeutic Recreation Journal*.

[28] Osborne H., Simpson J., Stokes G. (2010). The Relationship Between Pre-Morbid Personality and Challenging Behaviour in People With Dementia: A Systematic Review. *Aging & Mental Health*.

[29] Pocnet C., Rossier J., Antonietti J., Von Gunten A. (2013). Personality Traits and Behavioral and Psychological Symptoms in Patients at an Early Stage of Alzheimer’s Disease. *International Journal of Geriatric Psychiatry*.

[30] Von Gunten A., Pocnet C., Rossier J. (2009). The Impact of Personality Characteristics on the Clinical Expression in Neurodegenerative Disorders—A Review. *Brain Research Bulletin*.

